# Loss of Ezh2 promotes a midbrain-to-forebrain identity switch by direct gene derepression and Wnt-dependent regulation

**DOI:** 10.1186/s12915-015-0210-9

**Published:** 2015-11-30

**Authors:** Martina Zemke, Kalina Draganova, Annika Klug, Anne Schöler, Luis Zurkirchen, Max Hans-Peter Gay, Phil Cheng, Haruhiko Koseki, Tomas Valenta, Dirk Schübeler, Konrad Basler, Lukas Sommer

**Affiliations:** Institute of Anatomy, University of Zürich, Zürich, Switzerland; Friedrich Miescher Institute for Biomedical Research, Basel, Switzerland; Department of Dermatology, University Hospital Zurich, Zürich, Switzerland; RIKEN Center for Integrative Medical Sciences, RIKEN Yokohama Institute, Yokohama, Japan; Institute of Molecular Life Sciences, University of Zürich, Zürich, Switzerland

**Keywords:** Brain area identify, Epigenetics, Ezh2, Midbrain development, Neural stem cells, Wnt/β-catenin signaling

## Abstract

**Background:**

Precise spatiotemporal control of gene expression is essential for the establishment of correct cell numbers and identities during brain development. This process involves epigenetic control mechanisms, such as those mediated by the polycomb group protein Ezh2, which catalyzes trimethylation of histone H3K27 (H3K27me3) and thereby represses gene expression.

**Results:**

Herein, we show that Ezh2 plays a crucial role in the development and maintenance of the midbrain. Conditional deletion of *Ezh2* in the developing midbrain resulted in decreased neural progenitor proliferation, which is associated with derepression of cell cycle inhibitors and negative regulation of Wnt/β-catenin signaling. Of note, Ezh2 ablation also promoted ectopic expression of a forebrain transcriptional program involving derepression of the forebrain determinants Foxg1 and Pax6. This was accompanied by reduced expression of midbrain markers, including Pax3 and Pax7, as a consequence of decreased Wnt/β-catenin signaling.

**Conclusion:**

Ezh2 is required for appropriate brain growth and maintenance of regional identity by H3K27me3-mediated gene repression and control of canonical Wnt signaling.

**Electronic supplementary material:**

The online version of this article (doi:10.1186/s12915-015-0210-9) contains supplementary material, which is available to authorized users.

## Background

During organogenesis, cell proliferation, differentiation, and morphogenesis have to be tightly coordinated. This process involves extensive changes in gene expression, which entails epigenetic mechanisms such as DNA methylation, nucleosome remodeling, and post-translational modifications of the histones [1]. Epigenetic modifications mark the genome as regions that are either accessible or closed for the transcription machinery. Since these modifications can be inherited through cell divisions, epigenetic control is thought to maintain identity and behavior of a given cell type. Accordingly, fate switches associated, for instance, with the transition from a proliferative multipotent progenitor cell to a non-dividing terminally differentiated cell type, are accompanied and potentially controlled by changes in epigenetic information.

Key players in this process are polycomb group (PcG) proteins that form two complexes, polycomb repressive complex (PRC) 1 and PRC2, which repress gene activity by catalyzing trimethylation of lysine 27 on histone H3 (H3K27me3) [2, 3]. The catalytic subunit of PRC2 is the methyltransferase enhancer of zeste homolog 2 (Ezh2) or its homolog Ezh1 [4]. Ezh2 is essential for vertebrate development since mice lacking Ezh2 die around gastrulation [5]. A role of PcG proteins in the developing nervous system was suggested by experiments in embryonic stem cells undergoing neural differentiation, in which genes active during neurogenesis were shown to be dynamically marked by H3K27me3 and interference with demethylation of H3K27me3 prevented proper acquisition of a neural fate [1, 6, 7]. Conditional knock out (cko) of *Ezh2* in the developing murine forebrain around embryonic day (E) 10, i.e. before onset of neurogenesis, shifted the balance between self-renewal and differentiation of neural progenitors cells (NPCs) towards neuronal differentiation [8]. Similarly, the PRC1 component, Bmi1, was shown to control proliferation and self-renewal of NPCs during embryonic development by repressing the cell cycle inhibitor p21 [9]. However, at a later stage of cortical development, PcG proteins were reported to regulate the timely transition from neurogenesis to astrogenesis by repressing, among others, the proneural transcription factor Neurog1 [10]. These data reveal additional roles of Ezh2 during central nervous system development, apart from regulating stem cell properties. In support of this notion, downregulation of Ezh2 in NPC cultures derived from the forebrain at E14 promoted astrogenesis at the expense of oligodendrocyte development [11]. In contrast, conditional deletion of *Ezh2* in the neural crest did not affect stem cell proliferation and self-renewal nor timely neurogenesis and gliogenesis in the peripheral nervous system [12]. Together, these findings demonstrate that PcG proteins function in a cell type- and stage-dependent manner during neural development, presumably by repression of distinct sets of target genes.

To further address this issue, we conditionally deleted Ezh2 in the developing murine midbrain. Loss of Ezh2 resulted in drastically reduced growth of midbrain NPCs, which we found to involve derepression of specific cell cycle inhibitors as well as reduced canonical Wnt signaling. Moreover, *Ezh2* ablation in the midbrain led to derepression of a forebrain transcriptional program associated with reduced expression of midbrain markers. Thus, our study not only provides mechanistic insights on how NPC pool size is regulated in the midbrain, but also reveals a novel function of Ezh2 in consolidating regional identities in the developing brain.

## Results

### Conditional inactivation of *Ezh2* in the developing midbrain affects progenitor cell expansion

**Fig. 1:**
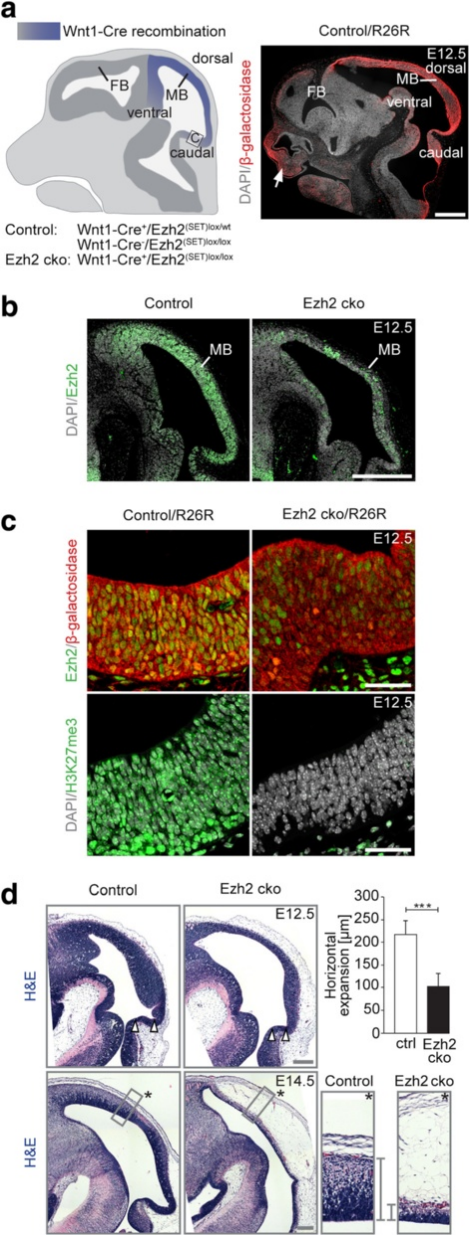
Wnt1-Cre-mediated Ezh2/H3K27me3 ablation affects midbrain expansion. (**a**) Left panel: scheme of the recombination area of the *Wnt1-Cre* line (indicated in blue) in the murine midbrain at E12.5. *Wnt1-Cre* ^*+*^ */Ezh2* ^*[SET]lox/wt*^ and *Wnt1-Cre* ^*−*^ */Ezh2* ^*[SET]lox/lox*^ mice are used as control while *Wnt1-Cre* ^*+*^ */Ezh2* ^*[SET]lox/lox*^ animals are referred to as Ezh2 conditional knock-out (cko). Right panel: Wnt1-Cre-driven recombination of the *R26R* reporter allele has been visualized by immunostaining against β-galactosidase, confirming recombination of the midbrain. Note that neural crest cells giving rise to craniofacial structures (white arrow) are also Wnt1-Cre recombined. (**b**) Immunostaining for Ezh2 reveals complete and partial ablation of Ezh2 protein in the caudal and rostral dorsal midbrain, respectively. (**c**) β-galactosidase immunostaining confirms full recombination of the caudal midbrain resulting in the absence of Ezh2 protein (upper panel) and the H3K27me3 repressive mark in the mutant (lower panel). (**d**) Hematoxylin and eosin staining (H&E) on sagittal midbrain sections at E12.5 (upper panel) and E14.5 (lower panel). Ezh2-deficient midbrains show reduced horizontal expansion in the inferior tectal region from E12.5 onwards (white arrowheads). n ≥3 in each group, ***P ≤0.001, Student’s *t*-test. Also, mutant neuroepithelium at E14.5 is thinner than the control highlighted by the grey brackets in high magnification pictures (asterisk indicates basal). Note that at E14.5 recombined neural crest-derived mesenchyme between neural epithelium and surface ectoderm is expanded in the mutant. DAPI staining serves as nuclear marker: **a** (right panel), **b**, **c** (lower panel); Scale bars: **a**, **b**, 500 μm; **c**, 40 μm; **d**, 200 μm; Error bars indicate SD; FB, Forebrain; MB, Midbrain; ctrl, Control

To address the role of Ezh2-mediated H3K27me3 in the developing midbrain, we conditionally deleted *Ezh2* in mice homozygous for the floxed allele of *Ezh2* using the *Wnt1-Cre* allele (Fig. 1a) [10, 12]. *Wnt1-Cre*^+^/*Ezh2*^[SET]^^*lox/lox*^ conditional knock-out (Ezh2 cko) mice survive to late developmental stages, but die around E18, displaying craniofacial abnormalities and heart malformations caused by concomitant activity of *Wnt1-Cre* in the neural crest [12]. In the midbrain, ablation of Ezh2 was evident from E10.5 onwards (Additional file 1: Figure S1). Of note, Ezh1 expression was very low in the embryonic midbrain and, importantly, was not affected upon conditional *Ezh2* inactivation (Additional file 1: Figure S1). Ezh2 loss was associated with widespread loss of H3K27me3, as shown by immunohistochemistry at E12.5 (Fig. 1b, c). Using the *ROSA26* Cre reporter allele driving β-galactosidase expression, we could also show full Wnt1-Cre-mediated recombination in the caudal midbrain [13]. Histological analyses revealed a marked reduction of the neuroepithelial thickness in the midbrain of *Ezh2* cko embryos at E12.5 as compared to normal embryos, which was even more pronounced at E14.5 (Fig. 1d). Furthermore, horizontal expansion of the neuroepithelium was decreased in mutant midbrains, as was most apparent in the isthmal and inferior tectal region at E12.5 (Fig. 1d).

**Fig. 2:**
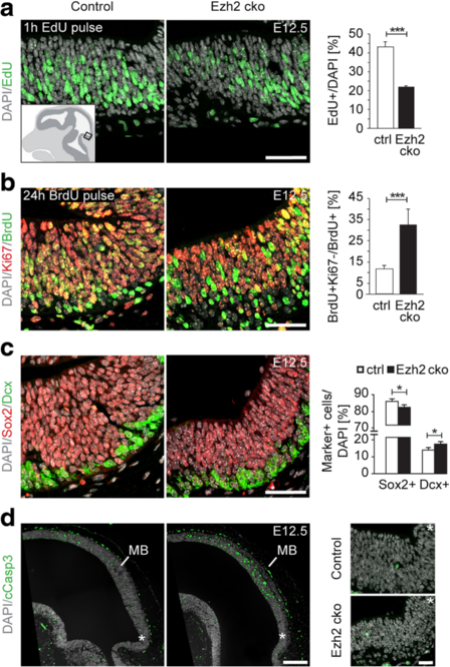
Ezh2-deficient neural progenitor cells (NPCs) show reduced proliferation and precocious cell cycle exit. (**a**–**c**) Confocal images of the inferior tectal midbrain at E12.5 with respective quantification. Cartoon insert indicates area of analysis for a–c. (**a**) Immunostaining against the thymidine analogue EdU after 1-h pulse labeling reveals reduced proliferation of NPCs in Ezh2-deficient cells. n ≥3 in each group, ***P ≤0.001, Student’s *t*-test. (**b**) After a 24-h BrdU pulse, staining against BrdU and the proliferation marker Ki67 distinguishes cells that have exited the cell cycle as BrdU-positive and Ki67-negative. Quantification of BrdU^ + ^Ki67^−^/BrdU^+^ cells demonstrates increased cell cycle exit of mutant NPCs. n ≥3 in each group, ***P ≤0.001, Student’s *t-*test. (**c**) Ezh2-deficient NPCs differentiate precociously as the quantification of Sox2-positive NPCs and Dcx-positive neurons show. n ≥3 in each group, *P ≤0.05, Student’s *t*-test. (**d**) Immunostaining for cleaved Caspase3 on sagittal midbrain sections reveals increased apoptosis in the dorsal midbrain of the mutant as compared to the control. In contrast, higher magnification confocal micrographs of the inferior tectal midbrain (indicated by the white asterisk) display no apoptosis. DAPI staining serves as nuclear marker for all images. Scale bars: **a**–**c**, 40 μm, **d**, 200 μm (left panel), 40 μm (right panel); Error bars indicate SD; ctrl, Control

These data are consistent with altered cell cycling of mutant neuroepithelial progenitor cells [14]. Indeed, the number of proliferative cells incorporating the thymidine analogue EdU during a 1-hour EdU pulse was significantly reduced in the developing midbrain of *Ezh2* cko embryos at E12.5 as compared to control littermates (Fig. 2a). The decrease of proliferative cells in the mutant midbrain could be associated with mutant neuroepithelial cells preferentially choosing to exit rather than to remain in the cell cycle. To address this possibility, we determined the fraction of Ki67-positive dividing cells after a BrdU pulse of 24 hours. Cells that had left the cell cycle were BrdU-positive but Ki67-negative, while cells that were still in the cell cycle at the time point of analysis were both BrdU- and Ki67-positive. At E12.5, a highly significant increase of cells exiting the cell cycle was detectable in the mutant as compared to the control (Fig. 2b). Immunohistochemistry for the NPC marker Sox2 and the differentiation marker Dcx further demonstrated that decreased proliferation in the midbrain of *Ezh2* cko embryos at E12.5 was accompanied by a reduction in the number of progenitor cells and a concomitant increase in differentiation (Fig. 2c). The increased neurogenesis in the *Ezh2* cko midbrain was also confirmed at E14.5 (Additional file 1: Figure S2). Cell survival was impaired in the dorsal rostral midbrain of mutant embryos but unchanged in the area used for quantification of mitotic cells (Fig. 2d). Thus, Ezh2 is essential for proper midbrain formation by controlling the pool size of NPCs.

### Ezh2 controls proliferation of neural progenitor cells by repressing cell cycle regulators and inhibitors of Wnt signaling

**Fig. 3:**
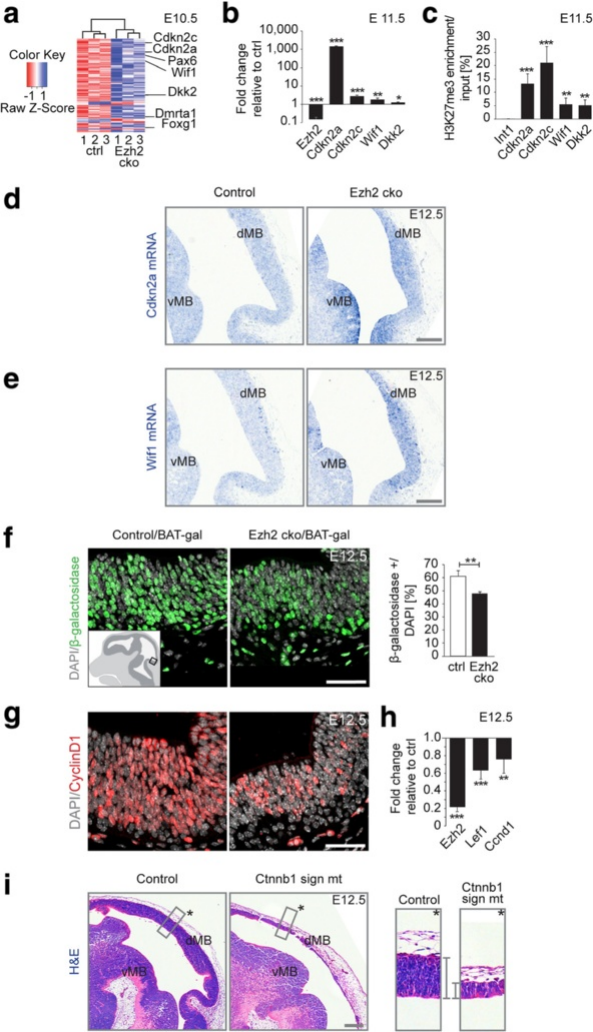
Neural progenitor cell proliferation is controlled by Ezh2-mediated repression of cell cycle and Wnt/β-catenin signaling inhibitors. (**a**) Microarray analysis of three dissected E10.5 control and mutant midbrains identified 126 differentially expressed genes (≥1.75×, *P* ≤0.01), the majority of which (114) are upregulated upon Ezh2 ablation. Genes further analyzed are indicated. (**b**) qRT-PCR for *Ezh2*, cell cycle regulators *Cdkn2a* and *Cdkn2c*, and Wnt signaling inhibitors *Wif1* and *Dkk2* on control and mutant E11.5 midbrains confirms microarray data. n ≥3 in each group, ***P ≤0.001, **P ≤0.01, *P ≤0.05, Student’s *t*-test. (**c**) Chromatin immunoprecipitation confirms the presence of H3K27me3 at the transcription start site (±500 bp) of *Cdkn2a*, *Cdkn2c*, *Wif1*, and *Dkk2*. Intergenic region *Int1* serves as unmethylated negative control. n ≥3 in each group, ***P ≤0.001, **P ≤0.01, Student’s *t*-test. (**d**–**e**) In situ hybridization for *Cdkn2a* (**d**) and *Wif1* (**e**) mRNA illustrates increased gene expression in Ezh2 mutants. (**f**) Immunostaining for β-galactosidase + cells on the BAT-*gal* Wnt/β-catenin signaling reporter line demonstrates diminished signaling in Ezh2-deficient midbrains. n ≥3 in each group, **P ≤0.01, Student’s *t*-test. Cartoon insert indicates area of analysis for **f** and **g**. (**g**) Immunostaining against CyclinD1 and qRT-PCR. (**h**) *Ccnd1* and *Lef1* Wnt signaling downstream targets show decreased expression upon Ezh2 ablation. n ≥3 in each group, ***P ≤0.001, **P ≤0.01, Student’s *t*-test. (**i**) H&E staining of E12.5 sagittal midbrain sections of controls and Wnt/β-catenin signaling-ablated embryos. Mutant embryos exhibit reduced neuroepithelium thickness indicated with grey brackets in the magnifications. DAPI staining serves as nuclear marker: **f**, **g**; Scale bars: **d**, **e**, 100 μm; **f**, **g**, 40 μm; **i**, 400 μm; Error bars indicate SD; ctrl, Control; dMB, Dorsal midbrain; vMB, Ventral midbrain

To identify the molecular mechanisms mediating Ezh2-dependent midbrain development, we used microarray analysis to compare the global gene expression patterns of control versus *Ezh2* cko cells isolated from the dorsal midbrain of E10.5 embryos. Cluster analysis of the transcriptome data indicated that the vast majority of differentially expressed genes were transcriptionally upregulated upon loss of *Ezh2* (Fig. 3a). This is consistent with the role of Ezh2 as a transcriptional repressor [15]. Gene ontology analysis of process networks revealed that differentially expressed genes were involved, among others, in negative regulation of proliferation, Wnt signaling, and cell cycle regulation (Additional file 1: Figure S3). Since misregulation of those processes very likely contributes to the described mutant phenotype, we focused our analysis on the aforementioned process networks. Among the genes derepressed in *Ezh2* cko cells, were the cyclin-dependent kinase inhibitors (*Cdkn*) 2a and 2c, which negatively regulate cellular proliferation [16–19]. Increased expression of *Cdkn2a* and *Cdkn2c* was also demonstrated by quantitative RT-PCR performed on midbrain cells isolated from E11.5 embryos (Fig. 3b). Moreover, in situ hybridization on sagittal sections of E12.5 control and mutant midbrains revealed the specific increase in expression of the cell cycle inhibitor *Cdkn2a* in *Ezh2* cko embryos (Fig. 3d). Finally, we performed an H3K27me3 ChIP assay on wildtype E11.5 midbrain cells and revealed that the promoters of *Cdkn2a* and *Cdkn2c* were occupied by H3K27me3. Thus, these cell cycle inhibitors appear to be direct targets of Ezh2-mediated epigenetic repression (Fig. 3c).

In addition, we also found other potentially relevant genes to be differentially expressed upon loss of *Ezh2.* In particular, inhibitors of the Wnt signaling pathway, such as *Wif1* and *Dkk2* [20, 21], were also significantly upregulated in *Ezh2* cko cells (Fig. 3a,b,e). H3K27me3 ChIP analysis confirmed that these Wnt signaling inhibitors appear also to be direct targets of Ezh2 activity (Fig. 3c). Canonical Wnt signaling has been demonstrated to control maintenance of midbrain neuroepithelial cells [22, 23]. Therefore, we investigated whether Wnt signal activity is indeed affected by loss of *Ezh2.* To this end, we made use of the *BAT-*gal Wnt signaling reporter allele, which monitors β-catenin activity by driving β-galactosidase expression in Wnt signaling-active cells [24]. In mice harboring this reporter allele, we observed a prominent reduction in the number of β-galactosidase-positive neural cells in the *Ezh2* cko midbrain at E12.5, as compared to the control (Fig. 3f). Accordingly, targets of Wnt signaling, such as *CyclinD1* and *Lef1*, were downregulated in the mutant midbrain, as shown by immunohistochemistry and quantitative RT-PCR, respectively (Fig. 3g,h).

Our data indicate that reduced canonical Wnt signaling might also contribute to the phenotype of *Ezh2* cko mice. To better understand the role of canonical Wnt signaling in regulating midbrain size, we took advantage of a mutant allele of the Wnt signaling component β-catenin (*Ctnnb1*^*dm/flox*^ referred to as *Ctnnb1 sign mt*) that disrupts Wnt/β-catenin-mediated transcriptional output but not cell-cell adhesion [22, 25]. While total loss of β-catenin leads to disintegration of the midbrain [22], loss of β-catenin signaling function did not affect the integrity of the neuroepithelium. However, very similar to *Ezh2* cko embryos (Fig. 1), the thickness and overall size of the midbrain was drastically reduced in *Wnt1-Cre*/*Ctnnb1 sign mt* cko mice at E12.5 (Fig. 3i). Therefore, Ezh2 appears to regulate the size of the developing midbrain both by direct repression of cell cycle inhibitors and, indirectly, by sustaining β-catenin signaling.

### Ezh2 represses forebrain identity in the developing midbrain

**Fig. 4:**
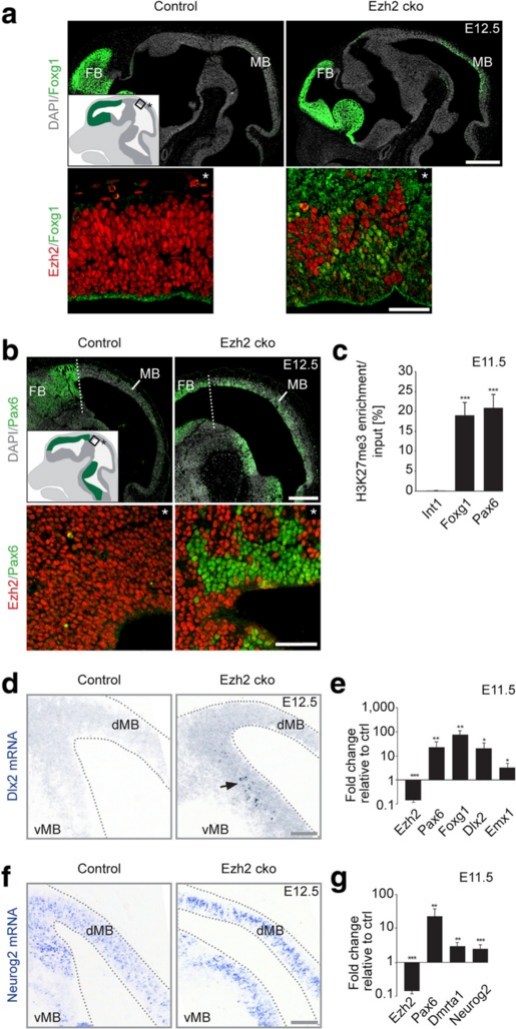
Establishment of forebrain identity in Ezh2-ablated midbrain cells. (**a**) Upper panel: antibody staining for forebrain-specific transcription factor Foxg1 on sagittal brain sections at E12.5 demonstrates forebrain-restricted *Foxg1* expression in the control and ectopic *Foxg1* expression in the dorsal mutant midbrain. The cartoon indicates regular *Foxg1* expression. Lower panel: high-magnification confocal images of immunostaining for Ezh2 and Foxg1 show ectopic Foxg1 expression in Ezh2-ablated cells (asterisk indicates basal). (**b**) Upper panel: antibody staining for Pax6 reveals ectopic *Pax6* expression in the *Ezh2* mutant midbrain. The cartoon inset illustrates regular *Pax6* distribution at E12.5 rostral of the di-mesencephalic boundary indicated by a white dotted line. Lower panel: immunostaining for Ezh2 and Pax6 reveals ectopic mosaic-like upregulation of Pax6 in Cre-recombined cells without Ezh2 (asterisk indicates basal). (**c**) ChIP confirms the presence of H3K27me3 repressive mark at the transcription start site of *Pax6* and *Foxg1* (±500 bp). The intergenic region *Int1* is unmethylated and serves as negative control. n ≥3 in each group, ***P ≤0.001, Student’s *t*-test. (**d**–**e**) In situ hybridization for *Dlx2* mRNA and qRT-PCR for *Foxg1*, *Pax6*, *Dlx2*, and *Emx1* demonstrate elevated expression of the forebrain transcription factors in Ezh2-deficient midbrains. n ≥3 in each group, ***P ≤0.001, **P ≤0.01, *P ≤0.05, Student’s *t*-test. Ectopic *Dlx2* expression is indicated by a black arrow (**d**). Downstream targets of *Pax6* – *Dmrta1* and *Neurog2* – are induced in mutant midbrains as shown by in situ hybridization for *Neurog2* mRNA (**f**) and qRT-PCR for *Dmrta1* and *Neurog2* (**g**). n ≥3 in each group, ***P ≤0.001, **P ≤0.01, Student’s *t*-test. DAPI staining serves as nuclear marker: **a**, **b**; Scale bars: **a**, **b**, 500 μm (upper panel); **a**, **b**, 40 μm (lower panel); **d**, **f**, 100 μm; Error bars indicate SD; ctrl, Control; FB, Forebrain; MB, Midbrain; dMB, Dorsal midbrain; vMB, Ventral midbrain

Intriguingly, the microarray analysis of control and *Ezh2* cko midbrain pointed to an additional set of Ezh2-regulated genes that are known to exhibit brain area-specific, rather than general cellular functions in the developing neuroepithelium. Notably, several forebrain specification genes were derepressed in *Ezh2* cko midbrains (Fig. 3a). In situ hybridization experiments, immunohistochemistry, and quantitative RT-PCR experiments were used to corroborate this finding. While normally Foxg1 is strongly expressed in the forebrain but absent in the midbrain, it was upregulated in the midbrain of *Ezh2* cko embryos at E12.5 (Fig. 4a). A quantitative analysis at E11.5 revealed a more than 75-fold induction of Foxg1 expression in the mutant midbrain (Fig. 4e). Likewise, the midbrain is normally devoid of Pax6 expression, whereas upon loss of Ezh2, Pax6 became broadly expressed in the midbrain, displaying a 22-fold induction at E11.5 (Fig. 4b,e). Furthermore, the forebrain markers Dlx2 and Emx1 were ectopically expressed in the midbrain of *Ezh2* cko embryos at E11.5 (Fig. 4d,e). However, when comparing mRNA levels of Ezh2, Pax6, Foxg1 and Emx1 in wildtype forebrain, wildtype midbrain, and Ezh2 cko midbrain of E12.5 embryos it became apparent that expression levels of ectopic forebrain markers in the mutant midbrain did not reach those of the forebrain (Fig. 4a; Additional file 1: Figure S4).

In most rostral regions of the dorsal midbrain, Ezh2-dependent H3K27me3 was only partially depleted in *Ezh2* cko embryos (Additional file 1: Figure S5). Incomplete *Wnt1-Cre*-mediated recombination was shown by tracking of recombined cells using the aforementioned *ROSA26* Cre reporter allele (*R26R*) [13]. Therefore, non-recombined cells were intermingled with clusters of Ezh2-deficient cells in the rostral midbrain of *Ezh2* cko embryos at E12.5 (Additional file 1: Figure S5). Strikingly, in this area, Ezh2 exhibited a perfectly inverse relationship with Foxg1 and Pax6 expression patterns, respectively, pointing to cell-autonomous mechanisms underlying the gain of forebrain markers in *Ezh2* cko midbrain cells (Fig. 4a,b). In support of this, H3K27me3 ChIP experiments performed with midbrain cells from control embryos at E11.5 demonstrated that the forebrain specification genes *Foxg1* and *Pax6* appear to be direct targets of Ezh2-mediated repression (Fig. 4c).

In the developing forebrain, *Pax6* acts upstream of the transcription factor Dmrta1, which itself regulates the expression of the proneural gene *Neurog2* [26]. Strikingly, *Pax6* upregulation in the *Ezh2* cko midbrain was associated with significant upregulation of both *Dmrta1* and *Neurog2* (Fig. 4f,g). Thus, although forebrain neuronal layer-specific markers could not be analyzed at later stages due to the substantial mass reduction and disturbed morphology of the mutant midbrain (Fig. 1d; data not shown), our data reveal the ectopic upregulation of a forebrain transcriptional program in the midbrain of *Ezh2* cko embryos.

### Ezh2 regulates midbrain identity by indirect mechanisms

**Fig. 5:**
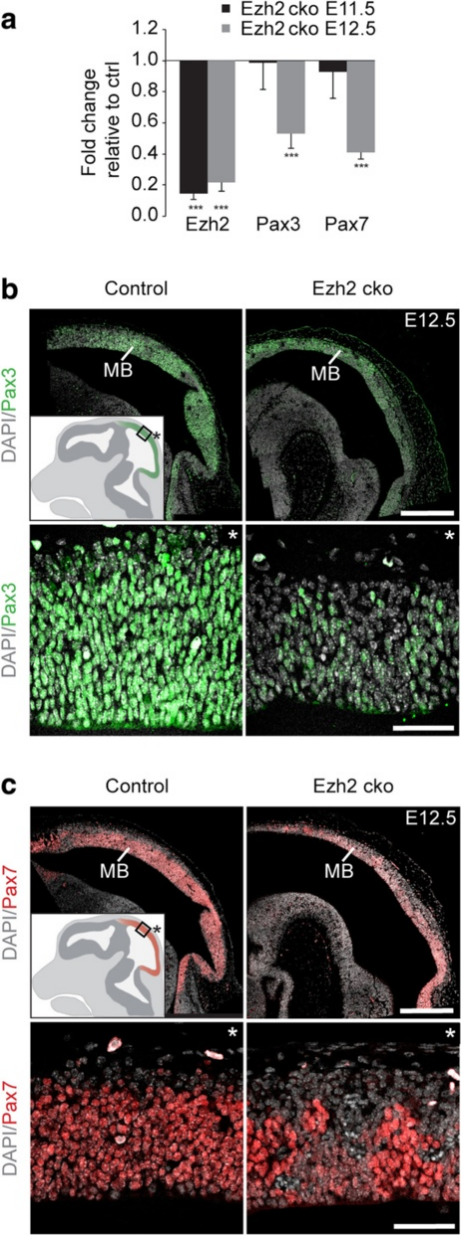
Ezh2 regulates midbrain identity indirectly. (**a**) qRT-PCR for *Ezh2*, *Pax3*, and *Pax7* on midbrain tissue isolated at E11.5 and E12.5 reveals a downregulation of midbrain transcription factors Pax3 and Pax7 in the absence of *Ezh2* after E11.5. n ≥3 in each group, ***P ≤0.001, Student’s *t*-test. (**b, c**) Immunostaining for Pax3 (**b**) and Pax7 (**c**) on sagittal midbrain sections shows diminished protein levels at E12.5 (magnification in lower panels, asterisk defines basal). The cartoon insets indicate regular *Pax3* and *Pax7* expression at E12.5, respectively. DAPI staining serves as nuclear marker: **b**, **c**; Scale bars: **b**, **c**, 500 μm (upper panel); **b**, **c**, 40 μm (lower panel); Error bars indicate SD; MB, Midbrain

Comparable to Pax6, Foxg1, Dlx2, and Emx1 in the developing forebrain, the transcription factors Pax3 and Pax7 have been shown to establish midbrain identity during vertebrate brain development [27, 28]. To address whether expression of these midbrain specification factors was also affected by loss of *Ezh2*, we performed quantitative RT-PCR and immunohistochemistry. While expression of Pax3 and Pax7 was unchanged at E11.5 as shown by qPCR, it was significantly downregulated at E12.5 (Fig. 5a). Consistent with these results, immunohistochemistry confirmed the presence of Pax3 at E11.5 (Additional file 1: Figure S6C) and the highly reduced expression of both transcription factors at E12.5 in *Ezh2* cko. Indeed, whereas Pax3 and Pax7 were detected in the entire dorsal midbrain neuroepithelium in control embryos, many cells in the mutant dorsal midbrain were devoid of Pax3 and Pax7 or showed reduced staining intensity (Fig. 5b,c). Thus, Ezh2-mediated H3K27me3 is required for proper expression of midbrain specification genes.

**Fig. 6:**
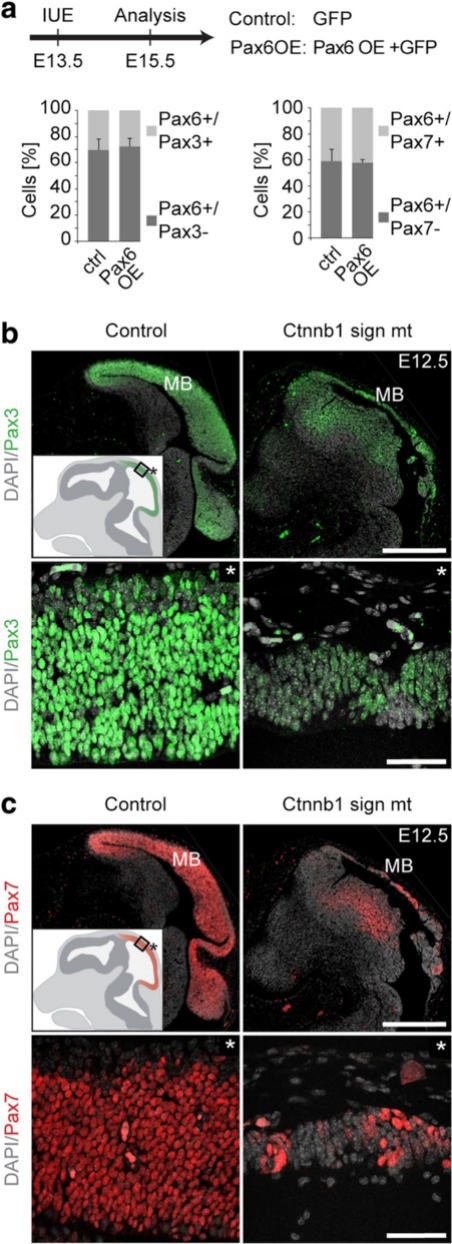
Midbrain markers are not directly repressed by Pax6 but are regulated indirectly by Wnt/β-catenin signaling. (**a**) A Pax6 overexpression construct together with a GFP-expressing vector (Pax6OE) or a GFP-expressing vector alone (Control) were delivered by in utero electroporation into the dorsal midbrain at E13.5. Two days later the proportion of GFP^+^ cells expressing midbrain markers Pax3 and Pax7 was analyzed. Overexpression of Pax6 does not affect the proportion of midbrain marker-expressing cells. n = 3 in each group, two different litters (also see Additional file 1: Figure S7B). (**b**, **c**) Immunostaining for Pax3 (**b**) and Pax7 (**c**) at E12.5 reveals decreased protein levels in midbrains with ablated Wnt/β-catenin signaling (Ctnnb1 sign mt) compared to control. Cartoon insets show regular expression pattern, asterisk indicates basal. DAPI staining serves as nuclear marker: **b**, **c**; Scale bars: **b**, **c**, 500 μm (upper panel); **b**, **c**, 40 μm (lower panel); Error bars indicate SD; IUE, in utero electroporation; ctrl, control; Ctnnb1 sign mt, Wnt/β-catenin signaling mutant; MB, midbrain

Our study identified the forebrain specification genes Foxg1 and Pax6 as targets of Ezh2 activity, which is in agreement with their increased expression in the *Ezh2* cko midbrain (Fig. 4). In contrast, the loss of midbrain identity markers in *Ezh2* cko embryos cannot be explained by direct Ezh2-mediated repression. In chicken embryos, overexpression of Pax6 has been reported to indirectly repress Pax3 and Pax7 expression in the trigeminal placode and at the forebrain-midbrain boundary, respectively [29, 30]. However, while we found Pax6 to be strongly upregulated in the *Ezh2* cko midbrain already at E11.5 (Fig. 4e), Pax3 and Pax7 were downregulated at E12.5 only (Fig. 5a), rather arguing against control of the midbrain specification factors by Pax6. To directly address this hypothesis, we performed in utero electroporation of a Pax6-overexpression vector together with a GFP expression vector. In parallel we electroporated the GFP expressing vector alone as a control. Monitoring GFP expression two days after in utero electroporation revealed the efficient targeting of the murine dorsal midbrain by this method (Additional file 1: Figure S7A). Coronal sections of electroporated midbrains were then used to quantify the number of Pax3- and Pax7-expressing cells per GFP-positive cells by immunofluorescence. For each condition, the midbrains of three embryos were electroporated and more than 800 cells were analyzed (Additional file 1: Figure S7B). However, as shown in Fig. 6a and Additional file 1: Figure S7C, ectopic expression of Pax6 did not influence Pax3 and Pax7 expression in dorsal midbrain cells. Hence, increased Pax6 expression is apparently unable to repress Pax3 and Pax7 in the established murine midbrain and is, therefore, unlikely the cause for downregulated expression of midbrain fate determinants in *Ezh2* cko embryos.

Previously, Wnt/β-catenin signaling was shown to activate Pax3 and Pax7 expression in the lateral neural plate and during neural tube closure [31–33]. Therefore, reduced Pax3/Pax7 expression in the *Ezh2* cko midbrain might be due to decreased canonical Wnt signaling in mutant brain tissue (Fig. 3). To investigate whether Wnt/β-catenin is required for expression of Pax3 and Pax7 in the dorsal midbrain, we performed immunohistochemistry on sagittal sections of *Wnt1-Cre*/*Ctnnb1 sign mt* cko embryos at E12.5. Loss of β-catenin signaling not only affected midbrain size, but also resulted in drastically reduced expression of both Pax3 and Pax7 (Fig. 6b,c). In fact, the midbrains of *Wnt1-Cre*/*Ctnnb1 sign mt* cko embryos displayed a phenotype very comparable to the one of *Ezh2* cko embryos (Fig. 5), with many mutant cells lacking Pax3 and Pax7 expression. Thus, the loss of midbrain identity markers in the *Ezh2* cko midbrain is apparently caused by indirect mechanisms, involving Ezh2-mediated control of canonical Wnt signaling.

## Discussion

**Fig. 7:**
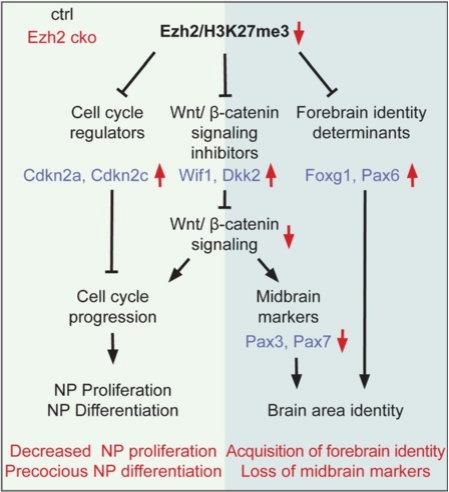
Proposed model for the role of Ezh2 in mouse midbrain development. Ezh2 controls neural progenitor proliferation and brain area identity via direct and indirect mechanisms. Ezh2 regulates cell cycle progression by H3K27me3-mediated repression of negative cell cycle regulators *Cdkna2* and *Cdkn2c* and Wnt/β-catenin signaling inhibitors *Wif1* and *Dkk2*. Also, Ezh2 maintains midbrain identity of cells by suppressing forebrain determinants Foxg1 and Pax6 and by maintaining Wnt/β-catenin signaling, which is essential for the expression of midbrain markers Pax3 and Pax7. Therefore, loss of Ezh2 in the developing mouse midbrain leads to decreased proliferation and precocious cell cycle exit of neural progenitors in addition to a partial loss of midbrain identity and ectopic establishment of forebrain identity

Epigenetic information can be passed from a dividing cell to its daughter cells, which is thought to support inheritance of specific gene expression patterns. In this way, epigenetic mechanisms supposedly consolidate cellular identities as, for instance, upon differentiation of a multipotent cell into a specific cell type [1]. In our study, we show that this mechanism is also involved in sustaining regional identity in the developing brain: Ezh2-mediated gene repression prevents midbrain cells from acquiring forebrain traits. Accordingly, conditional loss of *Ezh2* in midbrain NPCs not only affected their proliferation, but also resulted in derepression of a forebrain transcriptional program. We demonstrate that both the control of cell cycle progression and maintenance of regional identity involve direct H3K27me3-dependent gene repression as well as indirect mechanisms mediated, in particular, by modulation of canonical Wnt signaling (Fig. 7).

In many systems, Ezh2-dependent transcriptional control often guards the stem/progenitor state by repressing cell cycle exit and differentiation. This has also been shown for the developing forebrain, where Ezh2 regulates the transition from NPC proliferation to differentiation at a stage before overt neurogenesis [8]. In this previous study, loss of Ezh2 was associated with upregulation of several genes specifically expressed in differentiating cortical neurons. In contrast, expression of negative cell cycle regulators was normal or only slightly altered in the *Ezh2* cko forebrain [8]. Our work reveals that, similar to its role in the forebrain, Ezh2 is required for proper growth of the midbrain. Conditional *Ezh2* deletion resulted in drastically reduced numbers of proliferative midbrain NPCs, associated with elevated cell cycle exit and differentiation. Consistent with this phenotype, but different from the *Ezh2* cko forebrain [8], the cyclin-dependent kinase inhibitors *Cdkn2a* (also known as p16) and *Cdkn2c* (also known as p18) were directly regulated by Ezh2-mediated H3K27me3 in midbrain cells (Fig. 7). In addition, Wnt signaling inhibitors turned out to also be Ezh2 targets in the developing midbrain. Consequently, Wnt/β-catenin signal activity was significantly reduced upon loss of Ezh2. In the midbrain, Wnt1 is thought to maintain cells in a proliferative state [34, 35]. Accordingly, Wnt1 overexpression led to massive expansion of midbrain tissue [36]. In agreement with this earlier work, inactivation of the transcriptional output of Wnt signaling by means of a mutant β-catenin allele [22] impeded midbrain growth. Although the phenotype was more drastic when Wnt/β-catenin signaling was completely inactivated as opposed to only reduced in *Ezh2* cko embryos, our findings demonstrate how the regulation of a crucial signaling pathway by epigenetic repression contributes to the proper control of brain size (Fig. 7).

Surprisingly, apart from negative cell cycle regulators and Wnt signaling inhibitors, the forebrain determinants Pax6 and Foxg1 were also among the genes upregulated in the *Ezh2*-deficient midbrain neuroepithelium. Moreover, the promoters of both of these genes were marked by H3K27me3 in the midbrain. In embryonic stem cells, the Pax6 promoter is enriched for H3K27me3, which is removed when the cells acquire a forebrain neural fate [37]. Furthermore, Pax6 is one of the Ezh2-repressed genes in the murine heart [19]. However, Pax6 has not been identified before as an Ezh2 target in the context of the murine brain in vivo. We now show that Pax6 is derepressed in Ezh2-deficient midbrain cells from E10.5 to E12.5. During this timeframe, midbrain NPCs progress through the cell cycle at least twice [14], indicating that gene derepression mediated by Ezh2 loss is maintained through cell division. More importantly, we found Pax6 derepression in the *Ezh2*-deficient midbrain to be accompanied by activation of a forebrain transcriptional program that included Pax6, Foxg1, Dlx2, Emx1, and the transcription factors Dmrta1 and Neurog2, which themselves are downstream targets of Pax6 [26, 38]. Thus, Ezh2 represses a forebrain fate in the midbrain (Fig. 7). Presumably, additional factors would be required in an epigenetically derepressed midbrain to implement later forebrain features, such as characteristic layer formation and differentiation into specific neuronal subtypes. However, this cannot be achieved the *Ezh2* cko midbrain, because of the aforementioned other major role of Ezh2 in regulating midbrain growth.

Along with the gain of forebrain identity in Ezh2-deficient midbrain tissue, we observed a substantial reduction in the expression of dorsal midbrain markers Pax3 and Pax7, which are key regulators of midbrain development [27]. Unlike the changes in forebrain markers, reduced Pax3 and Pax7 expression was delayed upon loss of Ezh2 protein. Likewise, early expression of the midbrain patterning markers, Otx2, Fgf8, Pax2, and En2 [34, 39–41], was not affected in the midbrain lacking Ezh2 (Additional file 1: Figure S6A, B, D, E). Together with the initially normal expression of Pax3 and Pax7 in the *Ezh2* cko midbrain, our findings indicate that Ezh2 is not involved in the early establishment of midbrain identity, but rather in its maintenance.

The delayed timing of midbrain marker loss and the canonical function of Ezh2 as a transcriptional repressor suggested that Pax3 and Pax7 expression is indirectly controlled by Ezh2. Apparently, this did not involve Pax6-dependent downregulation of Pax3/7 as demonstrated by in utero electroporation of Pax6 in the specified dorsal midbrain. In chicken, Pax6 overexpression has been shown before to repress Pax3 and Pax7 expression in neural tissue [29, 30]. Since in these studies, Pax6-dependent regulation of Pax3/7 was indirect and mediated by a hitherto unidentified repressor, we propose that this repressing function might either be absent in the mammalian midbrain or at a stage when the Pax3/Pax7 expression domains have already been established. However, our work reveals an alternative mechanism for how midbrain-specific markers are being lost upon Ezh2 inactivation – indeed, reduced canonical Wnt signaling not only interfered with midbrain growth, but also with the continuous expression of Pax3 and Pax7 (Fig. 7). This finding was corroborated by the analysis of mutant midbrains, in which Wnt/β-catenin signaling was conditionally depleted.

## Conclusion

The change of brain area identities caused by loss of Ezh2 involves H3K27me3-mediated derepression of forebrain-specific transcription factors and indirect reduction of canonical Wnt signaling due to derepression of Wnt signal inhibitors. Thus, our study identifies epigenetic repression of multiple transcription factors and a central signaling pathway as a key mechanism in sustaining brain growth and regional identity.

## Methods

### Animal models

All animal experiments were conducted in accordance with Swiss guidelines and approved by the Veterinary Office of the Canton of Zurich, Switzerland. Previously described *Ezh2*^*[SET]lox/lox*^ mice [10] were crossed to *Wnt1-Cre* mice [42] to ablate Ezh2 function in the developing midbrain from E9.5. *Wnt1-Cre*^*+*^*/Ezh2*^*[SET]lox/lox*^ mice are referred to as Ezh2 cko while *Wnt1-Cre*^*−*^*/Ezh2*^*[SET]lox/lox*^ and *Wnt1-Cre*^*+*^*/Ezh2*^*[SET]wt/lox*^ littermates were used as control animals. All genotypes were present at Mendelian ratios and control animals showed no overt phenotype. Additionally, Ezh2 mice were crossed to a Cre-reporter line carrying the *R26RlacZ* allele [13] or to the canonical Wnt/β-catenin-signaling reporter mouse BAT-*gal* [24]. Also, floxed *Ctnnb1* mice were crossed to *Ctnn1*^*dm*^ [22, 25] under the Wnt1-Cre driver to generate mice with ablated Wnt/β-catenin signaling in the midbrain referred to as Ctnnb1 sign mt. All animals were bred on a C57/BL6 background. To generate embryos of a certain developmental stage, mice were mated overnight and the next morning was defined as E0.5.

### Staining procedures

Embryo heads or E15.5 brains were dissected, washed in PBS and fixed overnight in 4 % paraformaldehyde at 4 °C, followed by dehydration in ethanol and paraffin embedding. Sagittal or coronal 5-μm paraffin sections were deparaffinized, high-pressure antigen retrieval in citrate buffer (pH 6) was performed, and sections were subsequently stained following standard protocols. Primary antibodies used were mouse anti-Ezh2 (Cell Signaling Technology, #3147, 1:75), rabbit anti-H3K27m3 (Cell Signaling Technology, #9733, 1:500), chicken anti-β-galactosidase (Abcam, ab9361, 1:2000), rabbit anti-cleaved Caspase 3 (Cell Signaling Technology, #9661, 1:100), mouse anti-BrdU (Cell Signaling Technology, #5292 1:100), anti-Ki67 (rat Dako M7249, 1:50 and rabbit Abcam, ab15580, 1:200), rabbit anti-Dcx (Abcam, ab18723, 1:200), mouse anti-Sox2 (R&D, MAB2018, 1:100), mouse anti-CyclinD1 (Santa Cruz Biotechnology, sc-450, 1:200), anti-Pax6 (mouse DSHB, 1:50 and rabbit Covance, PRB-278P, 1:200), rabbit anti-Pax3 (Invitrogen, 38–1801, 1:100), mouse anti-Pax7 (DSHB Iowa, 1:100), chicken anti-GFP (Aves, GFP-1020, 1:300), rabbit anti-Pax2 (Zymed, 71–6000, 1:100), and rabbit anti-Foxg1 (Abcam, ab18259, 1:50). Secondary antibodies used were Alexa Fluor 546 goat anti-mouse IgG1 (Invitrogen, A-21123, 1:500), DyLight 488 goat anti-rabbit IgG (Jackson, 111-485-003, 1:500), DyLight 488 goat anti-chicken IgG (Jackson, 111-545-155, 1:500), and Cy3 goat-anti rabbit IgG (Jackson, 111-165-003, 1:500). Nuclei were stained with DAPI (Sigma, 1:1000).

For assessment of neural progenitor proliferation 30 mg/kg body weight thymidine analogue EdU was injected intraperitoneally (i.p.) into pregnant females 1 h before sacrificing the animals. Click-iT® EdU Alexa Fluor® 488 HCS Assay (life technologies, C10350) was used for visualizing incorporated EdU on brain sections. For cell cycle exit, 40 mg/kg body weight BrdU was injected i.p. into pregnant females 24 h before sacrificing the animals. For antibody staining against BrdU, high pressure antigen retrieval was followed by treatment of sections with 1 M HCl for 15 min at 25 °C and neutralization with 0.1 M sodium borate, pH 8.5, 2× 15 min. H&E was performed as previously described [43].

### In situ hybridization

Non-radioactive in situ hybridization with digoxigenin-labeled riboprobes was performed on paraffin sections. After deparaffinization and tissue treatment with 15–25 μg/mL proteinase K (Roche) for 5 min at 25 °C, a standard protocol was followed [44]. In situ probes for *Cdkn2a*, *Wif1*, and *Foxg1* were generated by in vitro transcription of PCR amplified genomic DNA fragments of about 500–900 bp. Primers used for PCR amplification are listed in Table S2 (Additional file 1: Table S2). In situ probes for Neurog2, Otx2, Fgf8, and En2 had been previously generated in the lab. John Rubenstein, USA, kindly provided the Dlx2 probe.

### Quantitative real-time PCR and microarray analysis

After tissue isolation from dorsal midbrains, total RNA was isolated with the RNAeasy kit (Qiagen) and RNase-Free DNase Set (79254, Qiagen) following the manufacturer’s instructions. For quantitative real-time PCR, 0.5 μg RNA were reverse transcribed with the Maxima First Strand cDNA Synthesis kit (Fermentas, K1641) and 1 μL of cDNA was used as input for quantitative real-time PCR. The reaction was carried out using LightCycler® SYBR Green I master mix (Roche, 4887352001) and was run on a LightCycler® 480 System (Roche). Each experiment was performed in a minimum of biological and technical triplicates. Obtained data were analyzed by the ∆Ct method and normalized to the expression levels of β-actin. Primers used are listed in Table S3 (Additional file 1: Table S3).

Isolated total RNA of E10.5 control (n = 3, from two different litters) and Ezh2 cko (n = 3, from two different litters) dorsal midbrains was used for microarray analysis performed at the Functional Genomics Center Zurich, Switzerland, using the Affymetrix A430 platform. The heat map in Fig. 3a and gene list in Table S1 (Additional file 1: Table S1) show differentially expressed genes with ≥1.75-fold change (*P* ≤0.01). Gene ontology network analysis was performed with MetaCore (Thomson Reuters). Obtained microarray data have been deposited in NCBI’s Gene Expression Omnibus [45] and are accessible through GEO Series accession number GSE74538.

### Chromatin immunoprecipitation (ChIP)

ChIP was performed as previously described [46] on chromatin prepared from dorsal midbrains of E11.5 NMRI embryos. A rabbit mab anti-H3K27me3 antibody (Cell Signaling Technology, #9733, 1:250) was used. Purified DNA (1 μL) was used as input for the quantitative real-time PCR and the reaction was carried out using LightCycler® SYBR Green I master mix (Roche, 4887352001), run on a LightCycler® 480 System (Roche). Primers were designed to amplify genomic DNA from a region flanking the transcriptional starting site ±500 bp and is devoid of local CpG islands. Primers used are listed in Table S4 (Additional file 1: Table S4). Obtained data were analyzed by the ∆Ct method and normalized to ChIP input. Also, intragenic region *Int1* (chr5: 79227331–79229070) is unmethylated and served as negative control.

### Plasmid preparation and in utero electroporation

In utero electroporation in mice was performed as previously described [47]. Briefly, after plasmid DNA was injected into the third ventricle of E13.5 brains, five electric pulses with a duration of 100 ms and an amplitude of 36 V at 400 ms intervals were applied to the dorsal midbrain with a pair of 3 mm diameter Tweezertrodes (BTX Harvard Apparatus, 45–0052) using the ECM Square Wave Electroporation System (BTX Harvard Apparatus, 45–0052). A Pax6 overexpression construct (pMF359-Pax6 plasmid, 2 μg/μL) together with a pCX-GFP plasmid (1 μg/μL) for the Pax6OE condition or a pCX-GFP plasmid (1 μg/μL) alone as control condition were introduced into wildtype (NMRI) embryos. Plasmids were amplified and purified using the Qiagen EndoFree Plasmid Maxi Kit following the manufacturer’s guidelines (Qiagen, 12362). Two days after electroporation, the embryos were recovered from the mother and E15.5 brains were dissected and further processed for immunohistochemistry. The pMF359-Pax6 vector was previously generated in the lab by cloning Pax6 with BamHI into a pMF359 vector kindly provided by M. Fussenegger. The pCX-GFP vector was a gift from O. Raineteau’s laboratory.

### Imaging, quantification, and statistical analysis

Epifluorescence and confocal images were taken with a Leica DMI6000 B or a CLSM Leica SP8 upright microscope, respectively, processed with Adobe Photoshop, and quantified manually using ImageJ. For all experiments and quantifications n ≥3 mutants and control embryos of at least two different litters were analyzed. For the quantification of immunostainings, at least three paraffin sections per embryo were analyzed. Representative images are shown in the figures. Measurement of the neuroepithelium thickness was done in ImageJ on H&E images. Statistical analysis was performed on Microsoft Excel using the unpaired, two-tailed Student *t*-test.

## Additional file

Additional file 1: Figure S1. (Related to Fig. 1) *Ezh2* expression is lost from E10.5. **Figure S2.** (related to Fig. 2) Ezh2 ablation results in increased neurogenesis. **Figure S3.** (related to Fig. 3) Gene ontology analysis. **Figure S4.** (related to Fig. 4) Expression levels of forebrain transcription factors in Ezh2 cko midbrain do not reach those of wildtype forebrain. **Figure S5.** (related to Fig. 4) Incomplete Cre-mediated recombination in the dorsal midbrain. **Figure S6.** (related to Fig. 5) Ezh2 ablation does not affect early midbrain patterning. **Figure S7.** (related to Fig. 6) Pax6 does not directly repress Pax3 and Pax7. **Table S1.** (related to Fig. 3) Differentially expressed genes of E10.5 control and Ezh2 cko midbrains. **Table S2.** Primers used for the generation of in situ probes by in vitro transcription. **Table S3.** Primers used for quantitative real-time PCR on embryo tissue samples. **Table S4.** Primers used for quantitative real-time PCR on DNA fragments isolated in H3K27me3 ChIP assay. (PDF 4989 kb)

## Abbreviations

β-gal: β-galactosidase
ChIP: Chromatin immunoprecipitation
cko: Conditional knock-out
Dcx: Doublecortin
E: Embryonic day
Ezh2: Enhancer of zeste homolog 2
H&E: Hematoxylin and eosin
H3K27me3: Trimethylation of histone H3K27
NPCs: Neural progenitor cells
PcG: Polycomb group
PRC: Polycomb repressive complex
