# Supplementary material for: Loss of Ezh2 promotes a midbrain-to-forebrain identity switch by direct gene derepression and Wnt-dependent regulation
Source: BMC Biol. 2015 Nov 30;13:103. doi: 10.1186/s12915-015-0210-9 (PMC4665911; doi:10.1186/s12915-015-0210-9)
Supplement: Additional file 1: Figure S1. — (Related to Fig. 1) Ezh2 expression is lost from E10.5. Figure S2. (related to Fig. 2) Ezh2 ablation results in increased neurogenesis. Figure S3. (related to Fig. 3) Gene ontology analysis. Figure S4. (related to Fig. 4) Expression levels of forebrain transcription factors in Ezh2 cko midbrain do not reach those of wildtype forebrain. Figure S5. (related to Fig. 4) Incomplete Cre-mediated recombination in the dorsal midbrain. Figure S6. (related to Fig. 5) Ezh2 ablation does not affect early midbrain patterning. Figure S7. (related to Fig. 6) Pax6 does not directly repress Pax3 and Pax7. Table S1. (related to Fig. 3) Differentially expressed genes of E10.5 control and Ezh2 cko midbrains. Table S2. Primers used for the generation of in situ probes by in vitro transcription. Table S3. Primers used for quantitative real-time PCR on embryo tissue samples. Table S4. Primers used for quantitative real-time PCR on DNA fragments isolated in H3K27me3 ChIP assay. (PDF 4989 kb) [file 12915_2015_210_MOESM1_ESM.pdf]

Additional file 1

Loss of Ezh2 promotes a midbrain-to-forebrain identity switch by direct gene derepression and Wnt-dependent regulation

Martina Zemke[1], Kalina Draganova[1], Annika Klug[1], Anne Schöler[2], Luis Zurkirchen[1], Max Hans-Peter Gay[1], Phil Cheng[3], Haruhiko Koseki[4], Tomas Valenta[5], Dirk Schübeler[2], Konrad Basler[5], Lukas Sommer[1*]

- **Figure S1** (related to Figure 1)
- **Figure S2** (related to Figure 2)
- **Figure S3** (related to Figure 3)
- **Figure S4** (related to Figure 4)
- **Figure S5** (related to Figure 4)
- **Figure S6** (related to Figure 5)
- **Figure S7** (related to Figure 6)
- **Table S1** (related to Figure 3)
- **Table S2**
- **Table S3**
- **Table S4**
- **Supplemental Figure Legends**

Figure S1

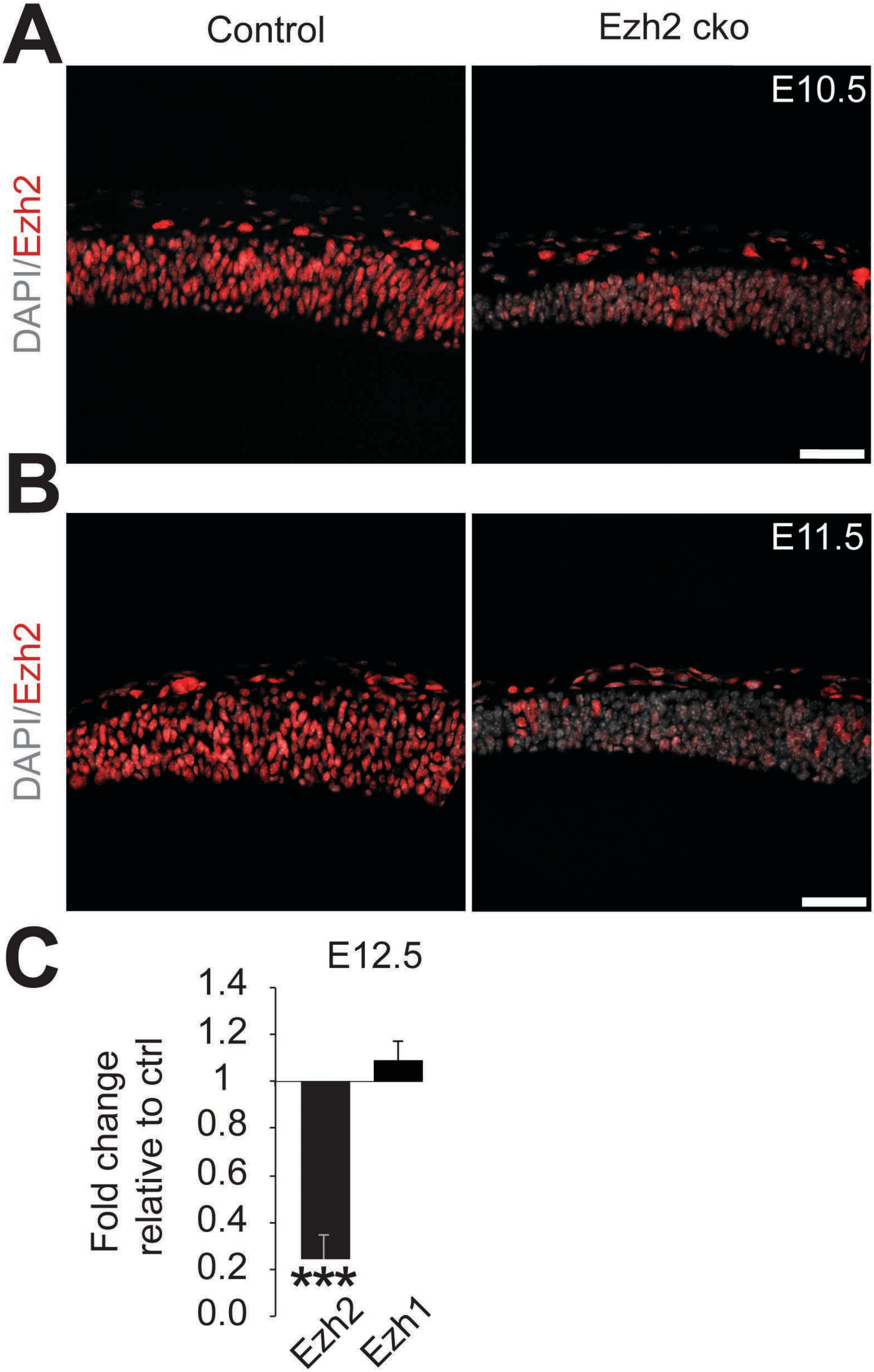
A
Control
Ezh2 cko
E10.5
DAPI/Ezh2
B
E11.5
DAPI/Ezh2
C
E12.5
1.4
1.2
1
0.8
0.6
0.4
0.2
0.0
Fold change relative to ctrl
***
Ezh2
Ezh1

Figure S2

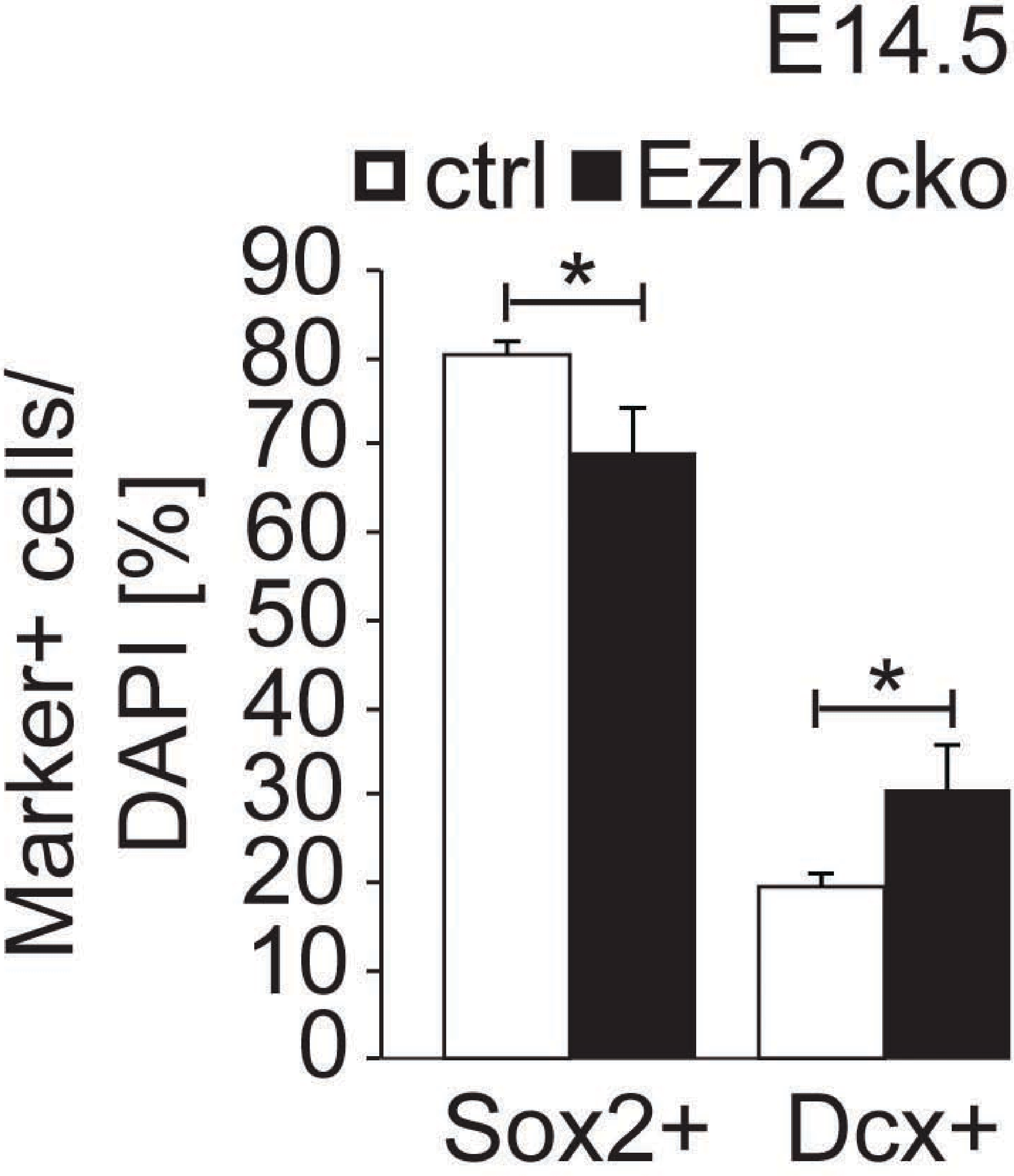
E14.5
ctrl
Ezh2 cko
Marker+ cells/
DAPI [%]
90
80
70
60
50
40
30
20
10
0
*
*
Sox2+
Dcx+

Figure S3

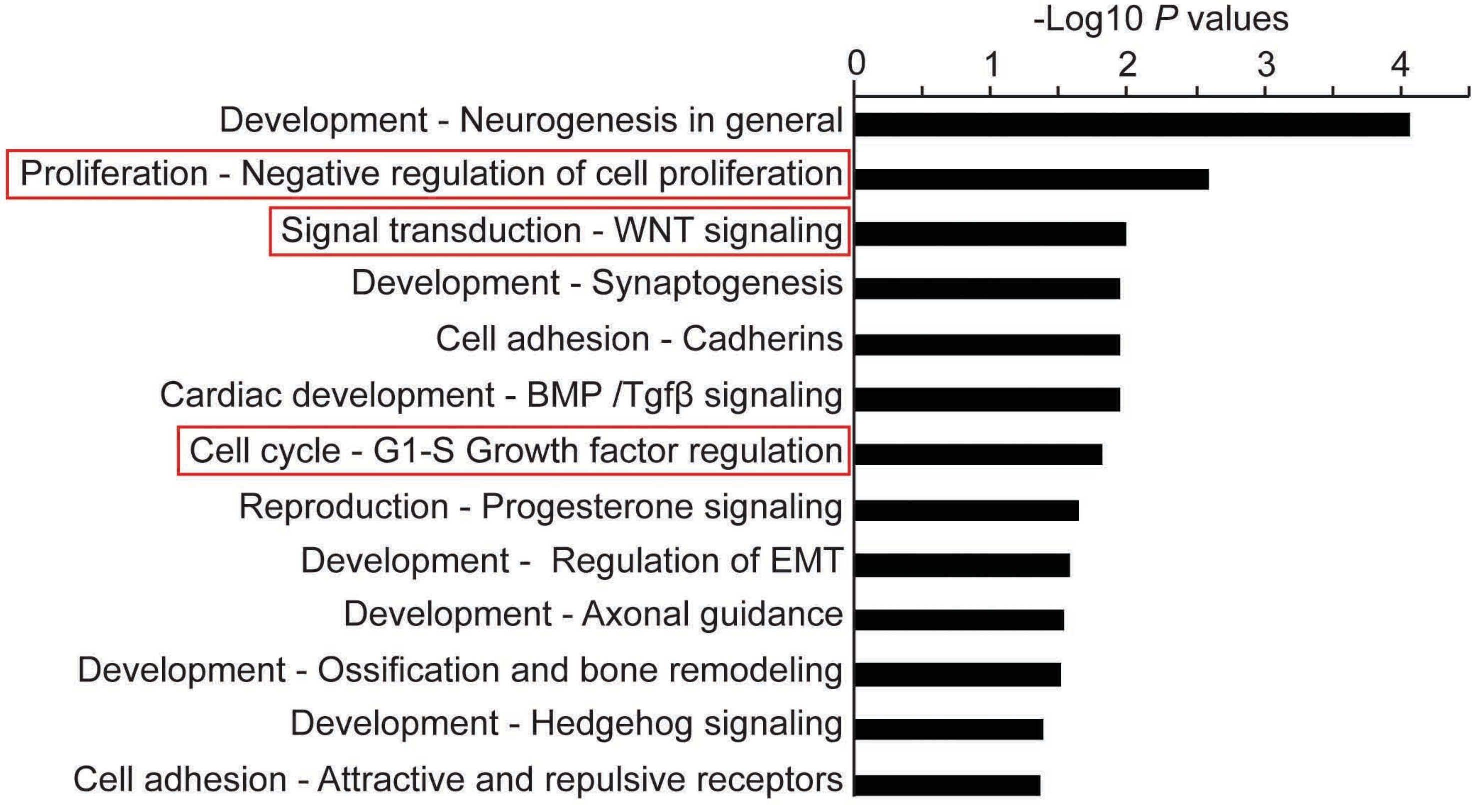

-Log10 P values
0
1
2
3
4
Development - Neurogenesis in general
Proliferation - Negative regulation of cell proliferation
Signal transduction - WNT signaling
Development - Synaptogenesis
Cell adhesion - Cadherins
Cardiac development - BMP /Tgfβ signaling
Cell cycle - G1-S Growth factor regulation
Reproduction - Progesterone signaling
Development - Regulation of EMT
Development - Axonal guidance
Development - Ossification and bone remodeling
Development - Hedgehog signaling
Cell adhesion - Attractive and repulsive receptors

Figure S4

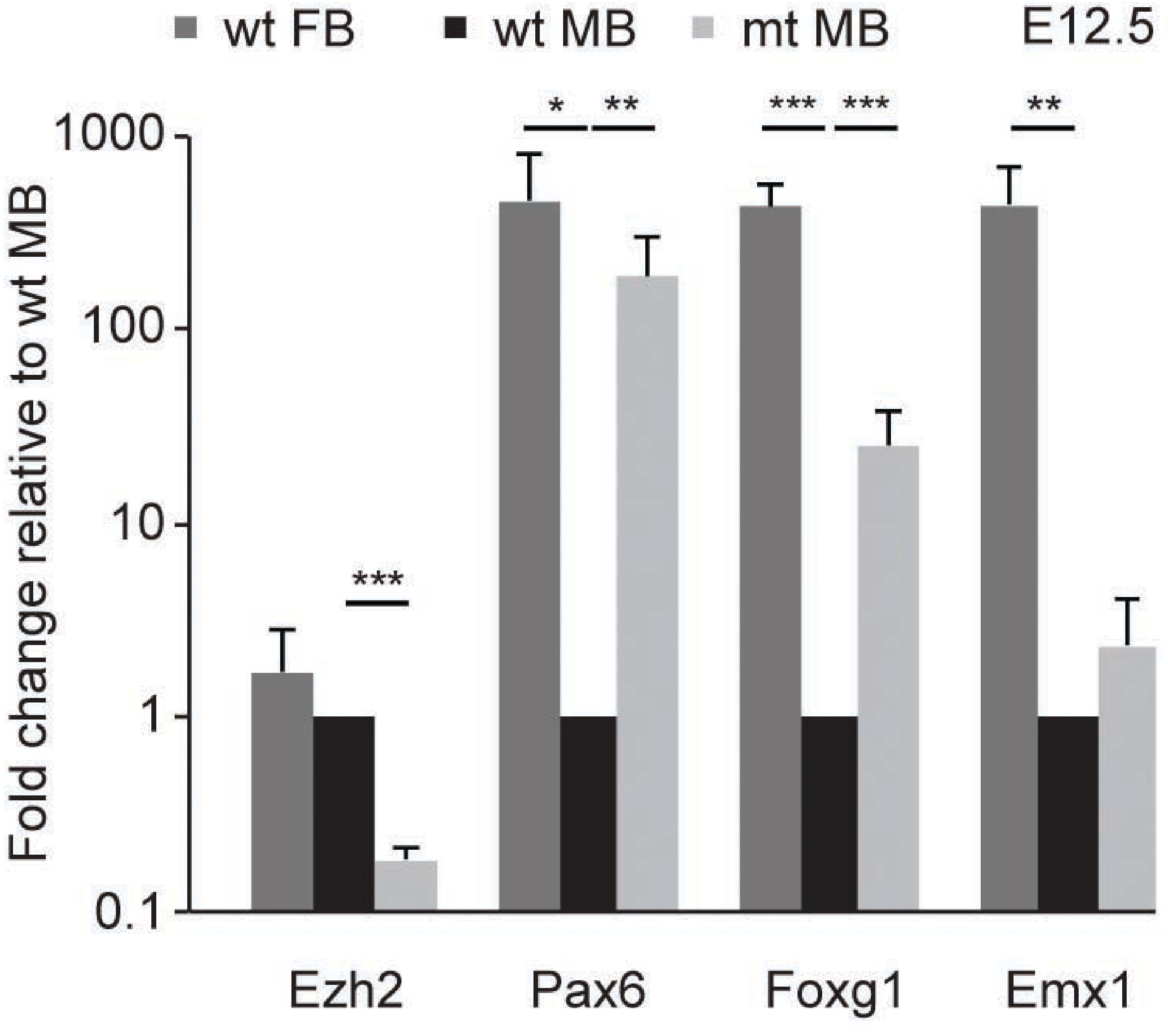
wt FB
wt MB
mt MB
E12.5
Fold change relative to wt MB
1000
100
10
1
0.1
*
**
***
Ezh2
Pax6
Foxg1
Emx1

Figure S5

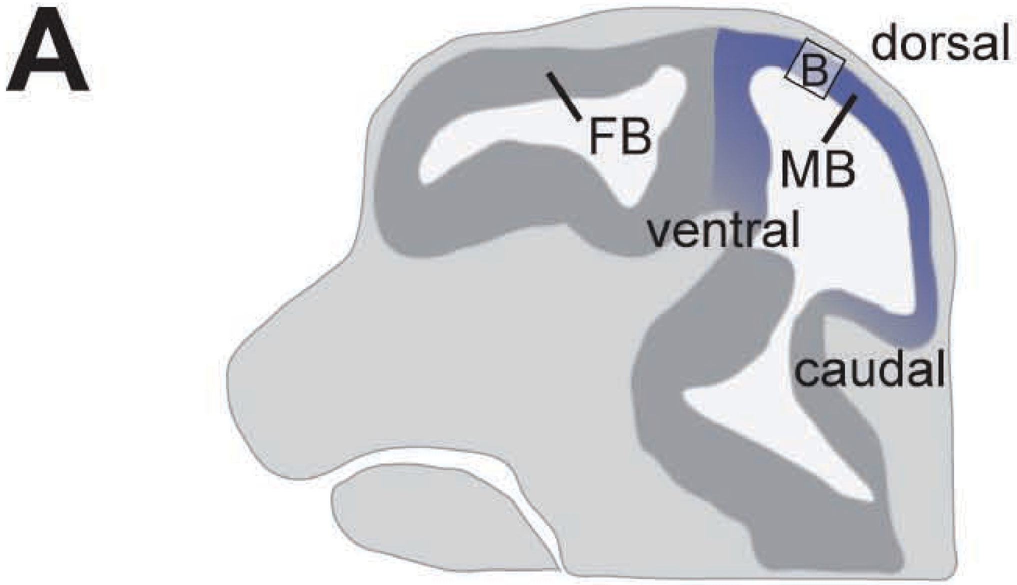
A
B
dorsal
FB
MB
ventral
caudal

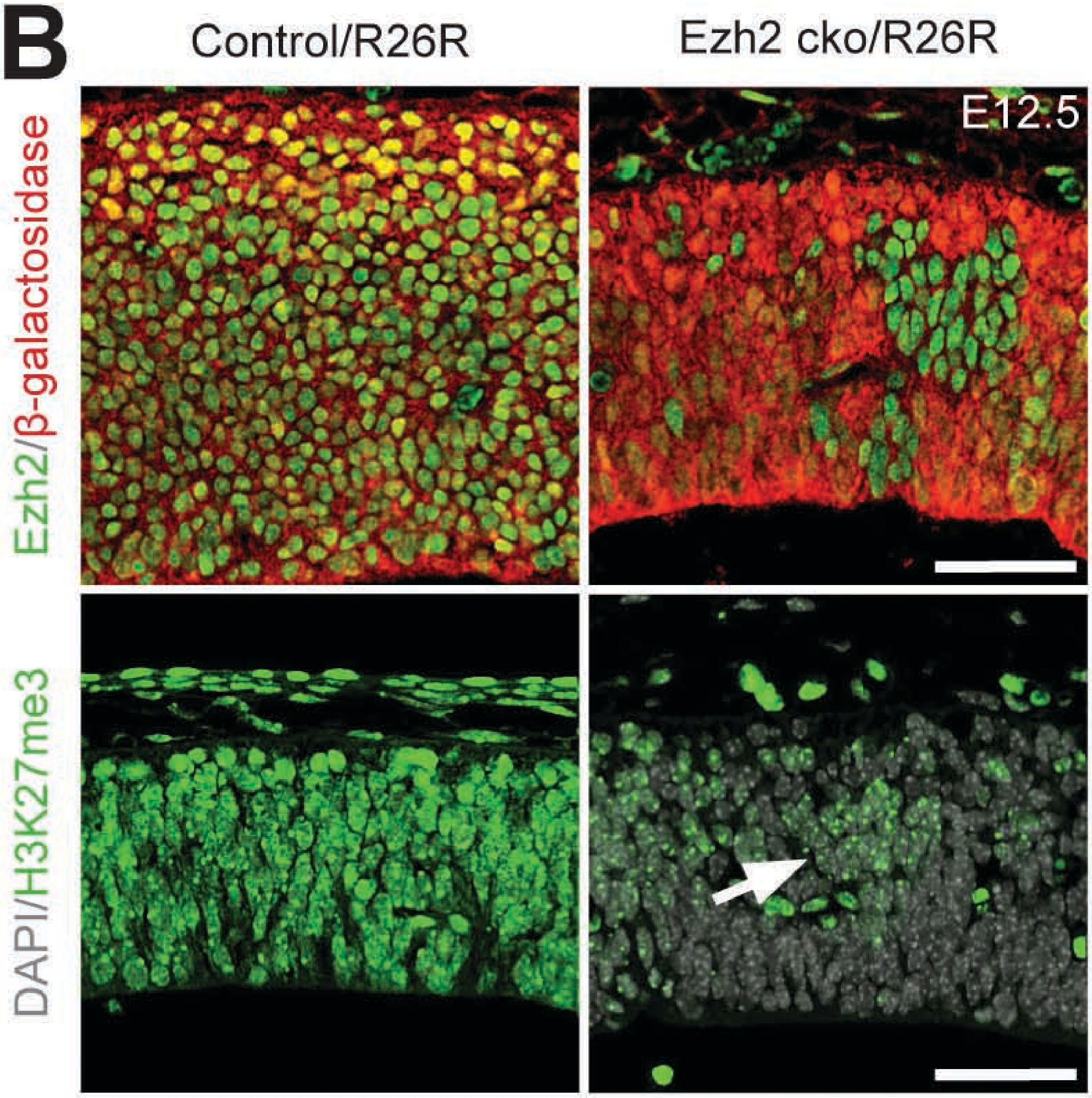
B
Control/R26R
Ezh2 cko/R26R
E12.5
Ezh2/β-galactosidase
DAPI/H3K27me3

Figure S6

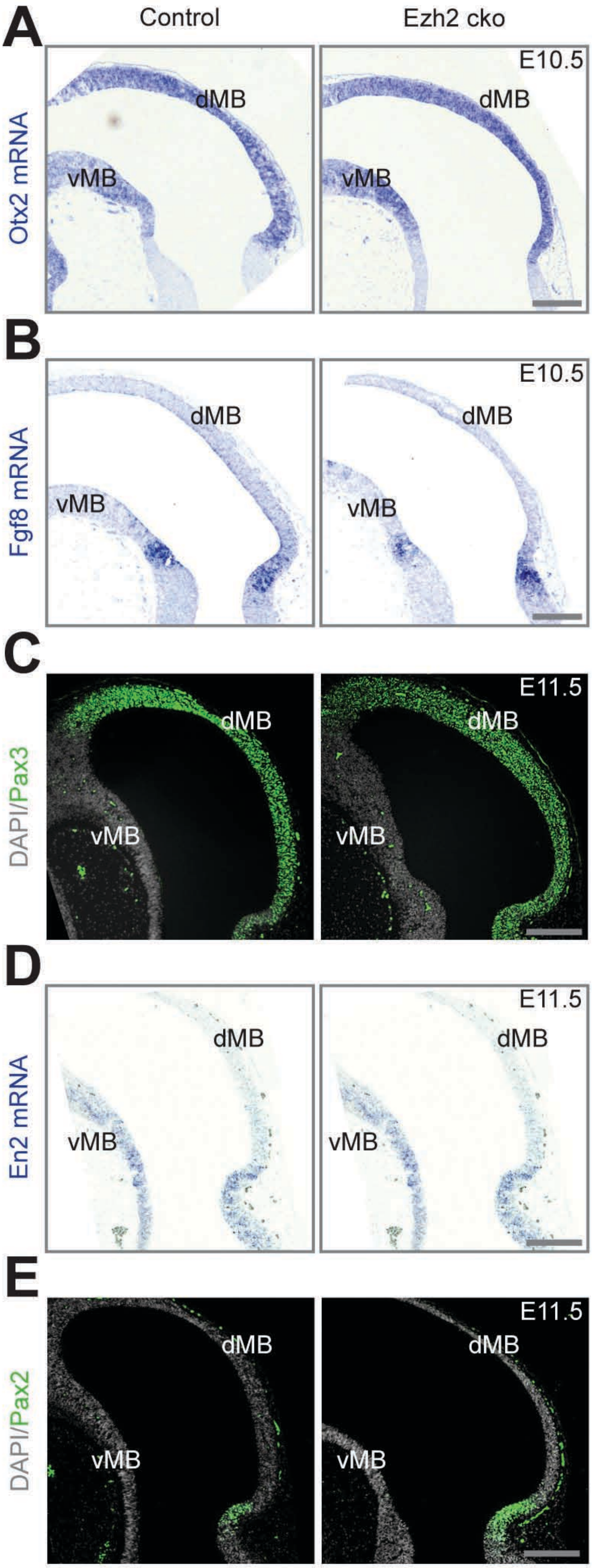
A
Control
Ezh2 cko
E10.5
Otx2 mRNA
dMB
vMB
B
E10.5
Fgf8 mRNA
dMB
vMB
C
E11.5
DAPI/Pax3
dMB
vMB
D
E11.5
En2 mRNA
dMB
vMB
E
E11.5
DAPI/Pax2
dMB
vMB

Figure S7

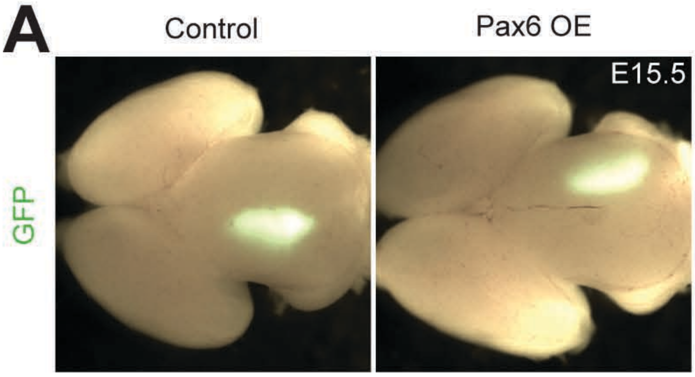

B

Control: GFP

Pax6 OE: Pax6 OE +GFP

n=3	total	GFP+/Pax3-	GFP+/Pax3+
control	942	311	631
Pax6 OE	865	253	612

n=3	total	GFP+/Pax7-	GFP+/Pax7+
control	1228	521	707
Pax6 OE	1022	411	611

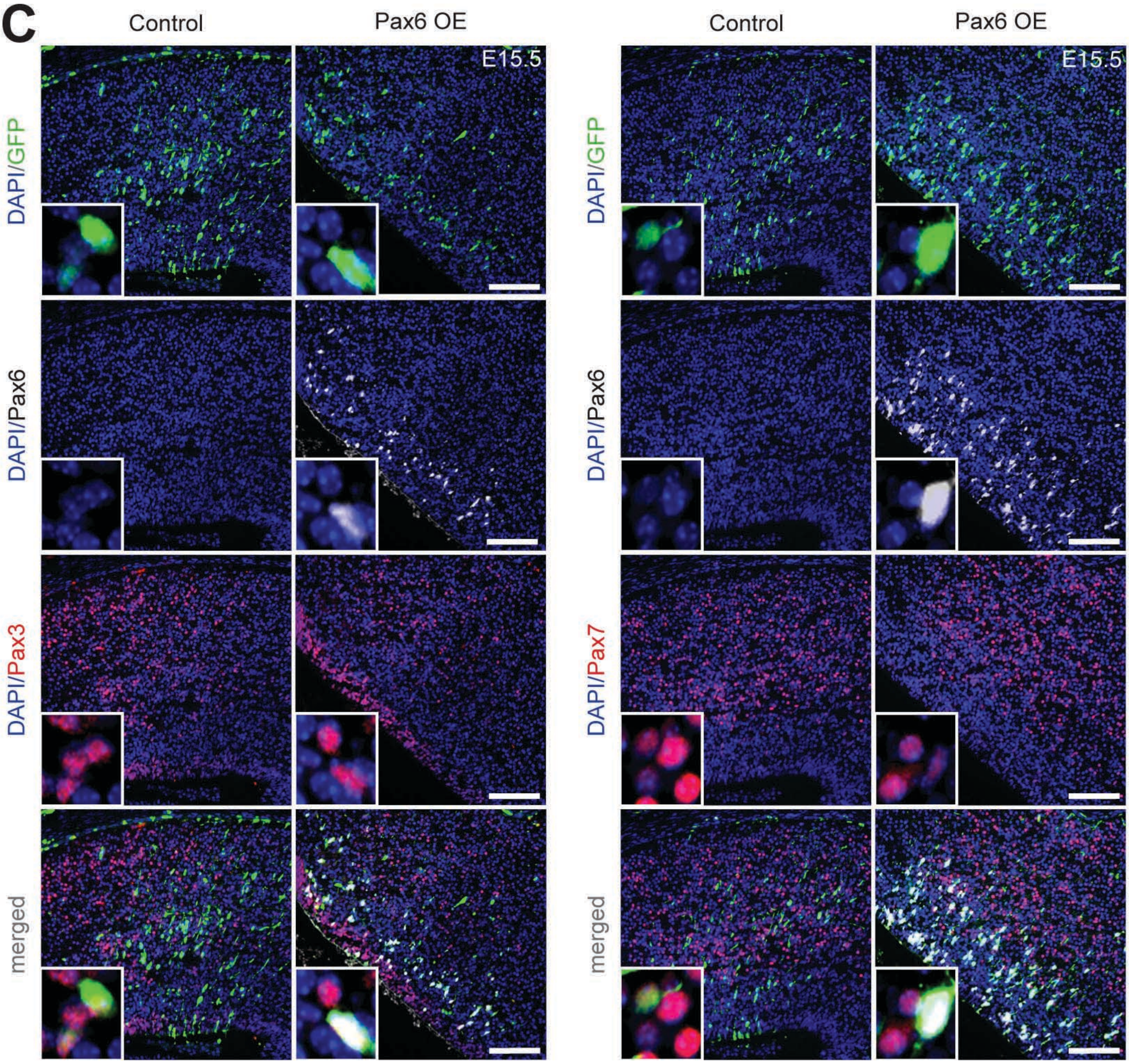

Table S1 (related to Figure 3). Differentially expressed genes of E10.5 control and Ezh2 cko midbrains.

Entrez GeneID	Observed probe	Gene	Expression Ratio (Ezh2 cko/control)
15228	1418357_at	Foxg1	11.44
15111	1436041_at	Hand2	5.25
14175	1420085_at	Fgf4	4.11
14175	1420086_x_at	Fgf4	3.83
18741	1424797_a_at	Pitx2	3.56
58801	1418203_at	Pmaip1	3.27
72388	1418488_s_at	Ripk4	3.02
14586	1433716_x_at	Gfra2	2.78
242523	1441579_at	Dmrta1	2.76
14012	1416236_a_at	Mpzl2	2.74
240041	1437128_a_at	Zfp945	2.72
12580	1416868_at	Cdkn2c	2.68
12578	1450140_a_at	Cdkn2a	2.67
69248	1430350_at	2610035F20Rik	2.66
245555	1440910_at	C77370	2.59
14807	1440177_at	Grik3	2.59
14765	1455498_at	Gpr50	2.59
14174	1441914_x_at	Fgf3	2.59
24117	1425425_a_at	Wif1	2.56
15423	1422870_at	Hoxc4	2.53
NA	1452493_s_at	NA	2.53
17075	1416579_a_at	Epcam	2.51
243771	1426774_at	Parp12	2.50
18260	1448873_at	Ocln	2.46
242620	1441107_at	Dmrta2	2.44
100039596	1439816_at	Tcf24	2.43
14174	1441350_at	Fgf3	2.42
15375	1418496_at	Foxa1	2.35
71653	1429055_at	4930506M07Rik	2.34
56811	1420512_at	Dkk2	2.31
12151	1448733_at	Bmi1	2.25
98363	1448507_at	Efhd1	2.24
353282	1434353_at	Sfmbt2	2.24
13837	1455426_at	Epha3	2.22
277353	1456515_s_at	Tcfl5	2.22
59012	1422643_at	Moxd1	2.21
211739	1452065_at	Vstm2a	2.20
18508	1419271_at	Pax6	2.19
54353	1460623_at	Skap2	2.16

100048658	1441045_at	Ddx43	2.14
18260	1440220_at	Ocln	2.11
104271	1420719_at	Tex15	2.10
12151	1417493_at	Bmi1	2.08
192198	1435832_at	Lrrc4	2.08
66892	1417978_at	Eif4e3	2.08
14394	1436889_at	Gabra1	2.04
15512	1417101_at	Hspa2	2.01
18933	1425526_a_at	Prrx1	2.01
66425	1452913_at	Pcp4l1	2.00
15874	1437323_a_at	Iapp	1.99
114332	1429379_at	Lyve1	1.99
319552	1441161_at	B230216G23Rik	1.98
12238	1433539_at	Commd3	1.98
209195	1454866_s_at	Clic6	1.98
18205	1434802_s_at	Ntf3	1.97
18997	1437588_at	Pou4f2	1.97
NA	1433578_at	NA	1.97
14815	1460303_at	Nr3c1	1.96
320405	1451499_at	Cadps2	1.95
193838	1460628_at	Eme2	1.95
12238	1454642_a_at	Commd3	1.95
329274	1457112_at	Fam163a	1.95
74499	1450179_at	Sost	1.94
12810	1423285_at	Coch	1.93
329828	1435417_at	AI464131	1.92
11847	1418847_at	Arg2	1.92
14560	1424007_at	Gdf10	1.90
18027	1456087_at	Nfia	1.90
12424	1419473_a_at	Cck	1.90
13176	1441572_at	Dcc	1.90
319887	1442156_at	E030030I06Rik	1.90
12931	1418476_at	Crlf1	1.89
71911	1426959_at	Bdh1	1.89
27226	1430700_a_at	Pla2g7	1.89
232174	1460011_at	Cyp26b1	1.88
633285	1443437_at	Rbm46	1.88
13176	1440487_at	Dcc	1.88
22239	1419063_at	Ugt8a	1.87
22408	1425377_at	Wnt1	1.87
100503463	1457042_at	AI256396	1.87
171211	1440162_x_at	Edaradd	1.86
19713	1436359_at	Ret	1.85
NA	1455538_at	NA	1.85
192198	1416097_at	Lrrc4	1.84
319211	1456387_at	Nol4	1.84

18028	1427680_a_at	Nfib	1.83
14025	1456632_at	Bcl11a	1.83
20908	1425530_a_at	Stx3	1.83
20464	1449967_at	Sim1	1.83
16011	1452114_s_at	Igfbp5	1.83
22773	1438737_at	Zic3	1.82
NA	1436203_a_at	NA	1.82
14025	1457072_at	Bcl11a	1.82
227753	1436991_x_at	Gsn	1.82
54353	1418895_at	Skap2	1.82
12293	1433643_at	Cacna2d1	1.81
245527	1440085_at	Eda2r	1.80
74342	1455883_a_at	Lrrtm1	1.80
12404	1423287_at	Cbln1	1.79
14077	1416023_at	Fabp3	1.79
16526	1449158_at	Kcnk2	1.79
24113	1450303_at	Vax2	1.79
16840	1460258_at	Lect1	1.79
12298	1436912_at	Cacnb4	1.79
110333	1438822_at	Rmst	1.79
102614	1448845_at	Rpp25	1.78
18027	1421163_a_at	Nfia	1.78
22773	1423424_at	Zic3	1.77
71911	1452257_at	Bdh1	1.77
58208	1435227_at	Bcl11b	1.77
171211	1437800_at	Edaradd	1.76
21808	1450923_at	Tgfb2	1.75
268345	1455258_at	Kcnc2	1.75
242509	1438861_at	Bnc2	1.75
18208	1454974_at	Ntn1	0.56
21350	1450517_at	Tal2	0.54
239618	1456512_at	Pdzrn4	0.54
100504141	1442377_at	Gm20083	0.54
77605	1436596_at	H2afv	0.54
74754	1418129_at	Dhcr24	0.53
69239	1425408_a_at	Pdzph1	0.50
215001	1427566_at	Wfikkn1	0.50
14912	1427420_at	Nkx6-2	0.39
56463	1416038_at	Snd1	0.36
330096	1438884_at	Shisa3	0.25
330096	1460000_at	Shisa3	0.21

Table S2. Primers used for the generation of *in situ* probes by in vitro transcription.

	Forward	Reverse
**Cdkn2a**	CCGTGTGCATGACGTG	AATTAACCCTCACTAAAGGGCATCGCTAGA AGTGAAGCTA
**Wif1**	TCAACTGTGACAAAGCAAAC	AATTAACCCTCACTAAAGGGATTGGCTTTAT CCGAAACCT

Table S3. Primers used for quantitative real-time PCR on embryo tissue samples.

	Forward	Reverse
**Ezh2(SET)**	GTGACCACAGGATAGGCATCT	CAAGGGATTTCCATTTCTCG
**Cdkn2a**	TCTGCTCAACTACGGTGCAG	ACGATGTCTTGATGTCCCCG
**Cdkn2c**	CCTTGGGGGAACGAGTTGG	AAATTGGGATTAGCACCTCTGAG
**Wif1**	TCTGGAGCATCCTACCTTGC	ATGAGCACTCTAGCCTGATGG
**Dkk2**	CTGATGCGGGTCAAGGATTCA	CTCCCCTCCTAGAGAGGACTT
**Foxg1**	AGCGACGACGTGTTCATCG	CCCGTTGTAACTCAA AGTGCTG
**Pax6**	CAGCTTGGTGGTGTCTTTGT	GCAGAATTCGGGAAATGTCG
**Lef1**	TGGTTAACGAGTCCGAAATCA	AGAGGACGGGGCTTGTCT
**Cnnd1**	CAGAGAGCTACAGACTCCGC	CGGTCGTTGAGGAGATTGGT
**Dlx2**	TCCTACTCCGCCAAAAGCAG	GGAGTAGATGGTGCGTGGTT
**Emx1**	CTAAGCGGGGTTTCACCATAGA	GCACTGGGGTGAGGATAGTTG
**Dmrta1**	CCTTTCCACGGTGACCTCAA	CATTTTCCAAGTCTGCGCGT
**Neurog2**	TCGTCAAATCTGAGACTCTGGAG	CGG CGC AGC TCC TCG TCC T
**Pax3**	CATTCGGCCTTGCGTCATTT	GTTGTCACCTGCTTGGGTTTG
**Pax7**	TGGTGGGGTCTTCATCAACG	CTTGCTGCCTCCGATAGCC

Table S4. Primers used for quantitative real-time PCR on DNA fragments isolated in H3K27me3 ChIP assay.

	Forward	Reverse
**Int1**	GCTCCGGGTCCTATTCTTGT	TCTTGGTTTCCAGGAGATGC
**Cdkn2a**	TAGCCTCAACAACATGTTCACGAAA	CAGGTTCTTGGTCACTGTGAGGATT
**Cdkn2c**	ATCTAAAACAACACTGCACAAAACA	AAAATGACGTACTTTGGAGAAACTG
**Wif1**	ATCAGGCTAGAGTGCTCATAGGTAA	CATAAGCCTCTAAATAGAGAATCCTGA
**Dkk2**	ACTCAACTCCATCAAGTCCTCTCTA	CAAAAGCTCTGATCCCTACCTCT
**Foxg1**	TAAGACCCTCTTTGCCAAGTTTTA	TGTAAAACGTTCACTTACAGTCTGG
**Pax6**	CTTTGAGAAGTGTGGGAACCAG	GGTTTCTATGACCTGAACACACAG

Supplemental Figure Legends:

Figure S1 (related to Figure 1). *Ezh2* expression is lost from E10.5. (A-B) Confocal images of antibody staining for Ezh2 at E10.5 (A) and E11.5 (B) show loss of Ezh2 protein from E10.5 onwards. **(C)** qRT-PCR for *Ezh2* and *Ezh1* on midbrain tissue isolated at E12.5 shows downregulation of *Ezh2* but no change of expression for its homolog Ezh1. DAPI staining serves as nuclear marker: B,C; Scale bars: 40 µm

Figure S2 (related to Figure 2). Ezh2 ablation results in increased neurogenesis. Ezh2-deficient NPCs show increased neurogenesis at E14.5 as demonstrated by quantification of Sox2-positive NPCs and Doublecortin-positive (Dcx) neurons. $n \geq 3$ in each group, $*P \leq 0.05$, Student's *t* test, Error bars indicate SD

Figure S3 (related to Figure 3). Gene Ontology (GO) analysis. Differentially expressed genes are involved in the indicated process networks represented by the -log10 *P* values.

Figure S4 (related to Figure 4) Expression levels of forebrain transcription factors in Ezh2 cko midbrain do not reach those of wt forebrain. Comparison of mRNA levels of Ezh2, Pax6, Foxg1 and Emx1 of wt forebrain, wt midbrain and Ezh2 cko midbrain at E12.5 show an upregulation of Pax6 and Foxg1 in mt midbrain compared to wt midbrain. Expression levels of forebrain transcription factors in mt midbrains do not reach those of wt forebrain, which are approximately 600x higher than in the wt midbrain. $n \geq 3$ in each group, 2 different litters. ***$P \leq 0.001$, **$P \leq 0.01$, *$P \leq 0.05$, Student's *t* test. Error bars indicate SD; FB, forebrain; MB, midbrain

Figure S5 (related to Figure 4). Incomplete Cre-mediated recombination in the dorsal midbrain. (A) Scheme of recombination area of the *Wnt1-Cre* line (indicated in blue) in the murine midbrain at E12.5. **(B)** Immunohistochemical detection of Ezh2 (upper panel) and H3K27me3 (lower panel) in dorsal midbrain area indicated in cartoon (A). Ablation of Ezh2 protein leads to a loss of the H3K27me3 repressive mark in the mutant. The Wnt1Cre-driven *R26R* reporter allele visualized by immunostaining against β-galactosidase (upper panel) shows partial Cre-mediated recombination in the dorsal midbrain. Areas that are not recombined still exhibit Ezh2 expression and show presence of H3K27me3 (white arrow). DAPI staining serves as

nuclear marker: B; Scale bars: B, 40 µm; Error bars indicate SD; FB, forebrain; MB, midbrain,

Figure S6 (related to Figure 5). Ezh2 ablation does not affect early midbrain patterning. (A-B) *In situ* hybridizations for *Otx2* (A) and *Fgf8* (B) mRNA show no difference in E10.5 midbrain patterning upon Ezh2 loss. **(C)** Also, antibody staining for the midbrain marker Pax3 reveals no change in distribution at E11.5. **(D-E)** Transcription factors important for the establishment of the mid-hindbrain-boundary are not changed upon Ezh2 ablation at E11.5 shown by *in situ* hybridization for En2 (D) and immunostaining against Pax2 (E). DAPI staining serves as nuclear marker: C, E; Scale bars: A, B, 100 µm; C, D, E, 200 µm; dMB, dorsal midbrain; vMB, ventral midbrain

Figure S7 (related to Figure 6). Pax6 does not directly repress Pax3 and Pax7. (A) Dissected E15.5 brains expressing GFP in the dorsal midbrain after *in utero* electroporation at E13.5. **(B)** Upper panel: A Pax6 overexpression construct together with a GFP vector (Pax6OE) or a GFP vector alone (Control) were delivered by *in utero* electroporation (IUE) into the dorsal midbrain at E13.5. Two days later brains were analyzed for the expression of midbrain markers *Pax3* and *Pax7*. Lower panel: Number of cells quantified for the different conditions are listed in the tables. n = 3 in each group **(C)** Representative images of coronal sections of electroporated midbrains used for quantification of GFP+ cells. Immunostaining for GFP, Pax6, Pax3 or Pax7

in combination with DAPI as nuclear marker. Insets show magnifications of single- or double-labeled cells of the respective stainings. Scale bars: 100 µm
